# Progress in Mirror-Based Fusion Neutron Source Development 

**DOI:** 10.3390/ma8125471

**Published:** 2015-12-04

**Authors:** A. V. Anikeev, P. A. Bagryansky, A. D. Beklemishev, A. A. Ivanov, E. Yu. Kolesnikov, M. S. Korzhavina, O. A. Korobeinikova, A. A. Lizunov, V. V. Maximov, S. V. Murakhtin, E. I. Pinzhenin, V. V. Prikhodko, E. I. Soldatkina, A. L. Solomakhin, Yu. A. Tsidulko, D. V. Yakovlev, D. V. Yurov

**Affiliations:** 1 Budker Institute of Nuclear Physics SB RAS, Lavrentyeva av. 11, Novosibirsk 630090, Russia; p.a.bagryansky@inp.nsk.su (P.A.B.); bekl@bk.ru (A.D.B.); A.A.Ivanov@inp.nsk.su (A.A.I.); E.Yu.Kolesnikov@inp.nsk.su (E.Y.K.); m.s.korzhavina@gmail.com (M.S.K.); olga.korobeynikova@yandex.ru (O.A.K.); A.A.Lizunov@inp.nsk.su (A.A.L.); v.v.maximov@inp.nsk.su (V.V.M.); s.v.murakhtin@inp.nsk.su (S.V.M.); e.i.pinzhenin@inp.nsk.su (E.I.P.); v.v.prikhodko@inp.nsk.su (V.V.P.); e.i.soldatkina@inp.nsk.su (E.I.S.); A.L.Solomakhin@inp.nsk.su (A.L.S.); D.V.Yakovlev@inp.nsk.su (D.V.Y.); dm.yurov@gmail.com (D.V.Y.); 2 Department of Physics, Novosibirsk State University, Pirogova str. 2, Novosibirsk 630090, Russia

**Keywords:** plasma physics, fusion neutron source, magnetic confinement, open magnetic trap

## Abstract

The Budker Institute of Nuclear Physics in worldwide collaboration has developed a project of a 14 MeV neutron source for fusion material studies and other applications. The projected neutron source of the plasma type is based on the gas dynamic trap (GDT), which is a special magnetic mirror system for plasma confinement. Essential progress in plasma parameters has been achieved in recent experiments at the GDT facility in the Budker Institute, which is a hydrogen (deuterium) prototype of the source. Stable confinement of hot-ion plasmas with the relative pressure exceeding 0.5 was demonstrated. The electron temperature was increased up to 0.9 keV in the regime with additional electron cyclotron resonance heating (ECRH) of a moderate power. These parameters are the record for axisymmetric open mirror traps. These achievements elevate the projects of a GDT-based neutron source on a higher level of competitive ability and make it possible to construct a source with parameters suitable for materials testing today. The paper presents the progress in experimental studies and numerical simulations of the mirror-based fusion neutron source and its possible applications including a fusion material test facility and a fusion-fission hybrid system.

## 1. Introduction

The most critical material issues in fusion energy development are caused by the intense irradiation of high energetic neutrons. The fusion reactor (DEMO, fusion power plant) cannot be built without performing an extensive program dedicated to the quality of materials to be used in the reactor core under high power irradiation by 14 MeV and secondary neutrons. For example, if the primary currently available materials for the first wall (ferritic-martensitic steels and vanadium alloys, which have a sufficiently long operating time) will be irradiated within 10 years with a neutron load of the first wall of 2 MW/m^2^, the level of radiation damage will achieve 200 dpa. In order to develop appropriate materials, which can withstand the high neutron load, a suitable neutron source with a flux density even higher than 2 MW/m^2^ is absolutely necessary as an irradiation facility for testing material samples. Since the beginning of the nuclear fusion program, various proposals for such a facility were made.

For a number of years, the Budker Institute of Nuclear Physics (Novosibirsk, Russia), in collaboration with other domestic and foreign organizations, has been developing a 14 MeV neutron source, which can be used for fusion material studies and for other application [1,2]. The projected neutron source of the plasma type is based on the plasma gas dynamic trap (GDT), which is a special magnetic mirror system for plasma confinement [3]. The GDT-based neutron source has a number of essential advantages. Research activity in the Budker Institute aims at completing the plasma-physics database for the proposed neutron source and demonstrating its feasibility and suitability by prototype GDT experiments.

The GDT-based neutron source (GDT-NS) could also be a candidate for fusion-driving sub-critical systems (FDS) dedicated to nuclear waste transmutation [4] and fission fuel breeding [5]. Such a plasma-based DT neutron source surrounded by a sub-critical fission blanket provides some advantages as compared to accelerator-driven systems. First of all, the presence of 14 MeV neutrons in the generated spectrum allows us to increase the efficiency of neutron production by (n, 2n) reactions, which is a threshold behavior, in the core region of the blanket. Moreover, the 14 MeV neutrons provide greater incineration/transmutation capabilities of the system, since it permits lower k_eff_-regimes. Finally, spatial distribution of the neutrons in the source opens new design possibilities for the fusion-fission system.

## 2. Recent Results of the GDT Experiment

**Figure 1:**
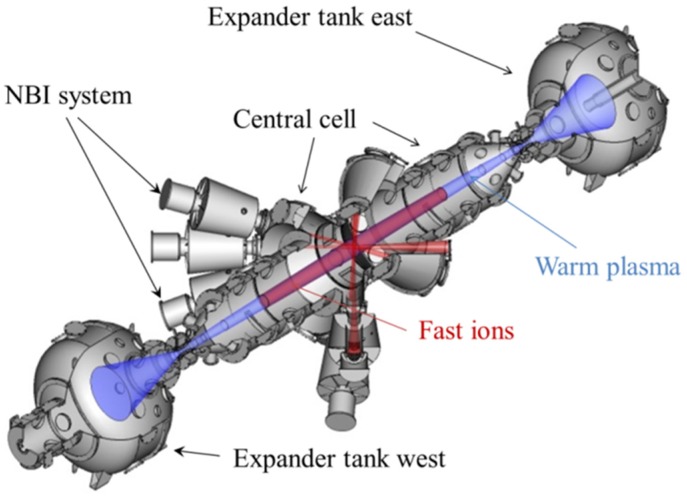
The layout of the GDT experiment.

Essential progress in plasma parameters was achieved in recent experiments at the GDT facility in the Budker Institute, which is a hydrogen (deuterium) prototype of the source [6]. Figure 1 shows the layout of the GDT experimental device with a quarter-section. The GDT is a 7-m-long axisymmetric mirror trap with a high mirror ratio (*B_0_* = 0.3 T, *B_m_* up to 15 T) for multicomponent plasma confinement. Warm maxwellian plasma is confined in a gas dynamic regime, which is characterized by collisional particle losses through the magnetic mirror into the end chambers of the device. An inclined 45° injection of eight deuterium atom beams (with energy of 20–25 keV and total power of 5 MW) produces fast ions oscillating back and forth between the hills of the magnetic field. The peaks of the fast ion density appearing near to their reflection points represent the volumes of high plasma pressure and intense fusion neutron production.

The GDT device is equipped by a set of modern diagnostics for measurement of different plasma parameters. Electron temperature and plasma density in the central cell are measured by a Tomson scattering system, which is similar to those in the GOL-3 device at the Budker Institute [7]. A relative plasma pressure value β can be estimated from the local magnetic field perturbation data measured by the beam-spectroscopic diagnostic based on the Motional Stark effect [8].

**Figure 2:**
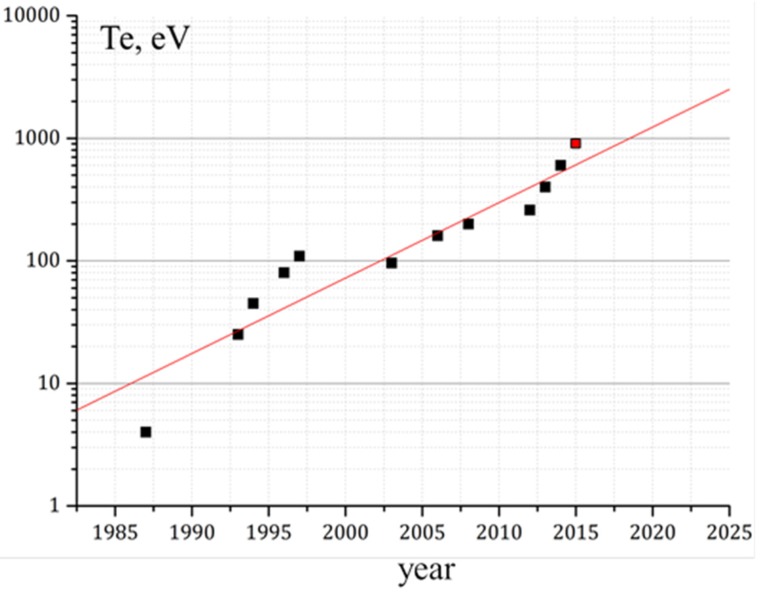
Electron temperature in GDT experiments *vs.* year of achievement. Red point is the achievement in year 2015.

Figure 2 presents the rise of the electron temperature in GDT experiments over the past 40 years. The maximal electron temperature obtained this year (red point) exceeds 0.9 keV which corresponds to the electron temperature in the first tokamaks. This result was achievable due to the additional ECR heating by a new ECRH system (two 54.5 GHz gyrotrons with 0.4 MW power each + beam lines) [9].

The electron temperature obtained in the GDT experiment substantially exceeds the previously predicted [2] limit for *T_e_* in a magnetic mirror trap with neutral beam injection: *T_e_* ~ 0.01 *E_inj_*, where *E_inj_* is the energy of the injected neutral atoms. It was established that under this condition the microturbulence is not excited in a mirror plasma. Thus, it is possible to abandon this prediction and use a self-consistent value of the electron temperature in GDT-NS modeling.

In GDT experiments the relative plasma pressure β exceeds 0.5, which corresponds to a high ion (deuteron) density up to 5 × 10^19^ m^−3^ with <*E_i_*> = 10 keV. All these results were obtained by using a new efficient method of transverse plasma confinement, the so-called “vortex confinement” [10]. Shear flows, driven via biased end plates and limiters, in combination with finite-Larmor-radius effects, are proven to be efficient in confining high-beta plasmas even with a magnetic hill on the axis.

Experimental achievements in GDT elevate the projects of a GDT-based neutron source on a higher level of competitive ability and make it possible to construct a source with reasonable parameters and suitable for materials testing today. The next sections of this paper are devoted to the numerical simulation of several neutron sources based on the achieved experimental data.

## 3. Simulation of Mirror-Based Fusion Neutron Source

### 3.1. Instruments and Tools

During the past few years several transport codes have been developed and applied for numerical studies of GDT-NS in parallel to the experimental research. The plasma physics calculations of the neutron source’s parameters have been performed by the *Integrated Transport Code System* (ITCS) [11]. The ITCS includes different numerical codes for plasma, particle transport, and neutron production. The main ITCS module is the three-dimensional (3D) *Monte Carlo Fast Ion Transport* (MCFIT) code. It is based on the theory of binary Coulomb collisions and the equations of classical magnetohydrodynamics. In the main scheme of the MCFIT code, which is standard for the Monte Carlo method, statistically independent histories of fast particles are generated and their contributions are summed up into well-defined estimation values for each parameter of interest. After simulating *N* particle histories, the final result for each parameter is calculated as an average with the statistical error determined by R.M.S. deviations. It is evident that the method converges as *N*^−1/2^. The transport code allows one to calculate a large variety of parameters. The result of the calculations is a database of parameters in the form of a discrete distribution on a phase space mesh defined by the user via a sequence of time intervals. The main parameters are as follows: the energy content of fast ions, the trapped NBI power, the charge-exchange loss power, the power of deceleration by electrons, the spatial distribution of nuclear fusion reactions (D–D and D–T), the neutron flux to specified spatial regions (“detectors”), and the distribution functions of fast ions over energy and pitch angle.

Last year, the MCFIT code has been substantially upgraded (to MCFIT+) for adequate simulation of various versions of the GDT-NS. For example, to calculate the energy distribution function of fast ions escaping from the trap through the magnetic mirrors, the code was supplemented with a special block for analyzing the energies of fast particles falling into the loss cone and reaching the mirror section. As a result, the absolute values of the fast ion flux were obtained for given energy intervals at the edge of each (western and eastern) mirror [12]. Also, the GDT-related physical phenomena (a vortex confinement, ambipolar plugging, magnetic field perturbation by high β, *etc.*) were taken into account during the code modification. The experimental and theoretical basis of these phenomena was obtained in the GDT experimental facility at the Budker Institute. At present, the ITCS with the main MCFIT+ code most completely describes the transport of fast ions in axisymmetric mirror traps and fusion devices based on them.

Brief simulations of GDT and GDT-NS plasma parameters, as well as optimization research, were done by the one-dimensional plasma code DOL. The DOL code was developed at the Budker Institute for the fast calculation of main plasma parameter evaluation in the mirror trap [13]. The DOL code is intended to calculate the dynamics of plasma processes in mirror traps with two-component plasma. In this code, the distribution function of fast ions is calculated by solving the bounce-averaged kinetic equation with allowance for variations in the fast-particle distribution function along the trap axis for the case of the time scale order *τ_ci_* « *τ_||_*« *τ_d_* ~ *τ_s_ ~ τ_ex_*. Here, *τ_ci_* is the time of cyclotron gyration; *τ_||_* is the bounce-oscillation period; and *τ_d_*, *τ_s_*, and *τ_ex_* are the characteristic times of deceleration, scattering, and charge exchange of fast particles, respectively. Longitudinal losses of warm plasma are modeled using a superposition of confinement mechanisms in the limiting cases by analogy with [14]. In particular, the possibility of plasma confinement in the gas-dynamic or weakly collisional case is taken into account and the effect of the ambipolar potential created by the population of fast particles is also allowed. In addition, the DOL code allows one to simulate the interaction of plasma with the neutral gas created as a result of hot atom injection. The correctness of the code operation is verified by comparing it with the data of GDT experiments and results of calculations by the MCFIT+ code.

### 3.2. Results of the Numerical Simulations

Table 1 presents a summary of simulation results for several projects of the mirror-based neutron source. The first column (a) shows achieved GDT experimental results for comparison with parameters of the projected devices. The GDT experimental parameters were also simulated by the numerical codes described above. Results of this simulation are in a good agreement with experimental data, which is the best proof of our numerical tools.

**Figure 3:**
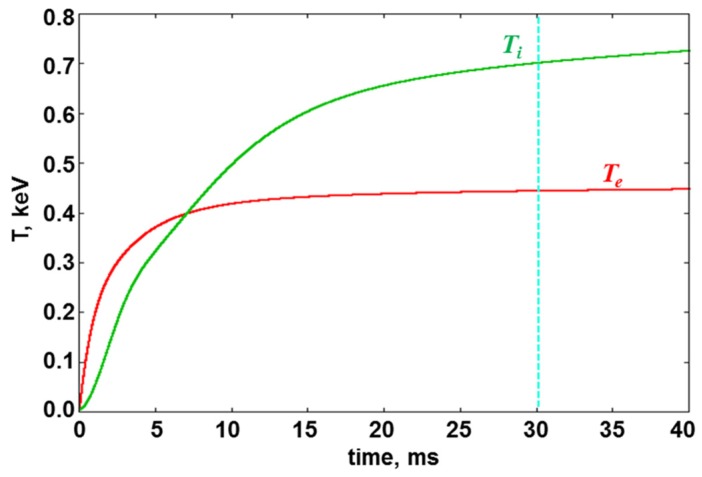
Simulated electron (*T_e_*) and ion (*T_i_*) temperatures in GDT-U *vs.* time after NBI is switched on.

The second column (b) presents parameters of the GDT-U project—a next step of the GDT experiments. A substantial modernization of the GDT magnetic system for 0.5 T in midplane and up to 15 T in mirrors is planned. A new NBI system with four modules of hydrogen or deuterium beams, each with 2.4 MW power, 20 keV energy and 30 ms pulse duration, will be realized in the near future. The main goals of the update are the demonstration of the increasing mirror plasma parameters closed to moderate the GDT neutron source project and achieving steady-state confinement. Figure 3 shows a projected time dependence of the ion (*T_i_*) and electron (*T_e_*) temperatures in GDT-U during NBI heating only. It seems that the 30 ms power beam injection is sufficient for a physical steady-state regime. An additional ECRH can increase the electron temperature over 1 keV at parameters listed in Table 1.

**Table 1:** Main results of neutron source simulations. (a) Achieved GDT experimental results; (b) GDT-U project—a next step of the GDT experiments; (c) GDT-based neutron source (GDT-NS) proposed for fusion material research; (d) Gas-Dynamic Multiple-mirror Trap (GDMT); (e) Optimized mirror-based NS.

Parameters	(a)	(b)	(c)	(d)	(e)
	GDT exp.	GDT-U (next)	GDT-NS	GDMT-NS	Mirror NS
Magnetic field, *B_0_*/*B_m_* (T)	0.34/12	0.5/15	1/15	1/9	2/15
Effective mirror ratio, *k*	35	30	15	75	7.5
Mirror-to-mirror distance, *L* (m)	7	7	10	10	14
NB injected/heat power, (MW)	5/3	9.6/7.2	40/30	40/30	100/90
NBI energy, *E_inj_* (keV)	25	20	65	65	80
Pulse duration, (s)	0.005	0.03	continuous	continuous	continuous
Warm ion density, *n_w_* (10^20^ m^−3^)	0.3	0.5	0.2	0.3	0.08
Fast ion density, *n_f_* (10^20^ m^−3^)	0.5	0.7	2.5	3.5	2
Mean ion energy, *T_i_* (keV)	10	10	35	30	60
Electron temperature, *Т_е_* (keV)	0.25/0.9 *	0.4/1*	0.7	1.5	6
Relative plasma pressure, β	0.6	0.5	0.5	0.5	0.5
Plasma radius, *a* (cm)	14	10	8	8	20
DT fusion energy gain factor, *Q_fus_*	–	–	0.05	0.1	0.5
DT fusion neutron power, *P_n_* (MW)	–	–	1.5	3	45
Neutron flux density, *q_n_* (MW/m^2^)	–	–	2	4	2

* With the additional ECRH.

**Figure 4:**
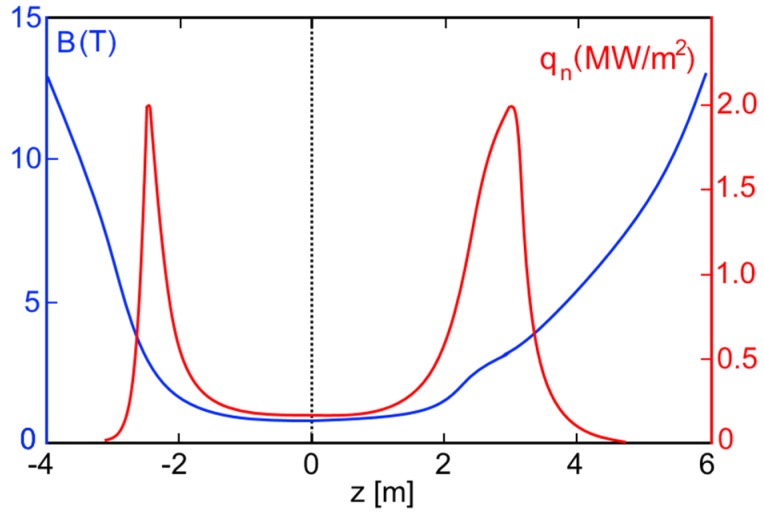
Magnetic field profile (blue, left scale) and neutron flux density profile (red, right scale) in GDT-NS.

**S**everal DT fusion neutron source projects were simulated on the basis of the achieved GDT experimental results (*T_e_* ~ 1 keV, β ~ 0.5). The first of them is the GDT-NS, which is proposed for fusion material research [2]. It is a 10--long axially symmetric mirror machine of the GDT type, with a magnetic field of *B_0_* = 1 T and a mirror ratio of 15. Similar to the GDT, the oblique injection of fast deuterium and tritium atoms into warm plasma produces a population of anisotropic fast deuterium–tritium (D–T) ions, which oscillate back and forth between the turning points near the end mirrors. As calculations show, there should be intensive radiation of 14 MeV neutrons in the vicinity of these turning points where the fast ion density has strong peaks. The source parameters are presented in column (c) of Table 1. The total power of fusion neutron production in this source is 1.5 MW which corresponds to a neutron flux density of about 2 MW/m^2^ in two effective testing areas of the order of 1 m^2^. It is important to note that quite a moderate energy of neutral beam injection is used in this version (65 keV). Magnetic field and neutron flux density profiles along the axis of the system are shown in Figure 4. The broadening of the right maximum of the flux density is determined by an appropriate tailoring of the magnetic field profile. A detailed description of the GDT-NS project, including SC coils, neutron shielding, test-zone design and other issues, is presented in [2].

**Figure 5:**
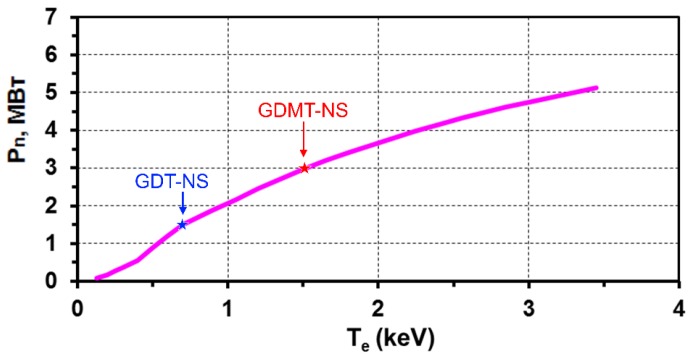
Fusion neutron power *vs.* electron temperature for the GDT-based neutron source with 40 MW 65 keV neutral beam injection. Stars correspond to GDT-NS and GDMT-NS parameters.

The electron temperature *T_e_* is a very important parameter for a plasma neutron source. The energy confinement time of fast ions in GDT-based mirrors (which is determined basically by the electron dragging), and hence the fusion neutron production in GDT-NS, is strongly dependent on *T_e_*. Figure 5 demonstrates the important role of the electron temperature and presents a rise of the fusion neutron power *vs. T_e_* in the GDT-based neutron source with a 40 MW 65 keV neutral beam injection (see column (c) and (d) of Table 1). One can see that even greater than 4 MW neutron power can be achieved in GDT-NS with moderate injection energy of a D-T mixture if the electron temperature will be higher than 10^−2^ *E_inj_* and reaches the value of 2–3 keV.

The Gas-Dynamic Multiple-mirror Trap (GDMT) [15] combines the features of existing GOL-3 [16] and GDT devices, namely the GDT-like central cell with sloshing ions produced by intense neutral beam injection, and the multiple-mirror end sections to suppress axial plasma losses. Such a combination became feasible due to recent findings in both GOL-3 and GDT experiments. The GDMT-based neutron source has an improved axial confinement with the effective mirror ratio *k = k_m_*∙χ up to 100, where *k_m_* = *B_m_/B_0_* is the magnetic mirror ratio, *B_m_*, *B_0_* are magnetic field values in the mirror and central cell, and χ is a multiplication factor related to multiple-mirror confinement. The simulated GDT-NS parameters (see column (d) in Table 1) allow us to propose this neutron source as a basis for different applications including a fusion material test facility with up to 4 MW/m^2^ neutron flux density (see Figure 5) and moderate fusion-driven (hybrid) systems (FDS).

**Figure 6:**
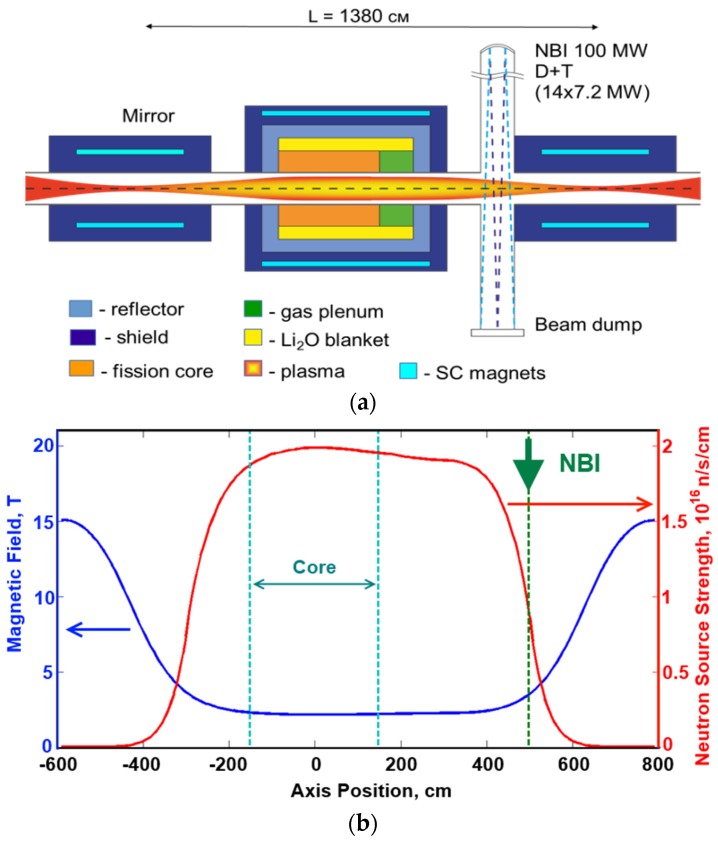
(**a**) Schematic view of FDS with mirror NS; (**b**) Magnetic field strength (**left scale**) and neutron emission yield (**right scale**) along the NS axis. Vertical dashed lines indicate the sub-critical core and the neutral beam injector positions.

The first analysis of possibility to use the GDT-based neutron source as a driver in the sub-critical system (FDS) was made in [4], and the necessity of optimizing the GDT-NS parameters was shown. Optimized mirror-based NS with *Q_fus_* = 0.5 uses 100 MW of 80 keV NBIs and has a 6-m-long neutron zone (n-zone) with a production up to 1.4 × 10^19^ n/s. It assumes a kinetic regime of axial confinement, vortex transverse confinement, and warm maxwellian plasma minority for the DCLC stabilization. Main parameters of optimized Mirror NS are presented in column (e) of Table 1. This powerful NS is proposed as a basis of future FDS (hybrids) for MA burning or other nuclear energy applications. An approximate layout of the mirror-based FDS for minor actinide (MA) incineration is presented in Figure 6a. The fusion source is assumed to be used with a sub-critical fission MA core and a Li_2_O-filled tritium breeding blanket. The magnetic field strength and neutron emission yield (as numbers of neutrons per second per axial length unit) along the NS axis are shown in Figure 6b. The neutron flux density on the first plasma wall was limited by the value of 2 MW/m^2^, so the plasma wall radius is 30 cm. Further reduction of the first wall load is possible by increasing of the first wall radius. This is simply achieved in an axially symmetric chamber design.

## 4. Conclusions

Recent experimental results of the GDT show the possibility of realizing competitive neutron sources based on an axisymmetric mirror cell.

The next step of GDT experiments with an updated device (GDT-U) at the Budker Institute was proposed and numerically simulated. The results show the possibility of achieving steady-state confinement with high plasma parameters.

Fusion neutron sources based on GDT and multi-mirror GDMT were numerically simulated. Proposed neutron sources with *Q_fus_* up to 0.1 can be used as a basis for fusion material test facilities and moderate fusion-driven systems (FDS).

Numerical optimization of the mirror-based neutron source for the driver in the FDS hybrid system was done. The proposed source with *Q_fus_* = 0.5 can be used for effective MA burning in FDS or for other applications in nuclear energy.
